# Co-bedding of Preterm Newborn Pigs Reduces Necrotizing Enterocolitis Incidence Independent of Vital Functions and Cortisol Levels

**DOI:** 10.3389/fped.2021.636638

**Published:** 2021-04-01

**Authors:** Anders Brunse, Yueming Peng, Yanqi Li, Jens Lykkesfeldt, Per Torp Sangild

**Affiliations:** ^1^Comparative Pediatrics and Nutrition, Department of Veterinary and Animal Sciences, Faculty of Health and Medical Sciences, University of Copenhagen, Copenhagen, Denmark; ^2^Department of Neonatology, Shenzhen People's Hospital (The Second Clinical Medical College, Jinan University; The First Affiliated Hospital, Southern University of Science and Technology), Shenzhen, China; ^3^Nordic Bioscience Clinical Development A/S, Herlev, Denmark; ^4^Experimental Animal Models, Department of Veterinary and Animal Sciences, Faculty of Health and Medical Sciences, University of Copenhagen, Copenhagen, Denmark; ^5^Department of Neonatology, Rigshospitalet, Copenhagen, Denmark; ^6^Hans Christian Andersen Children's Hospital, Odense, Denmark

**Keywords:** necrotizing enterocolitis, skin-to-skin care, co-bedding, hydrocortisone, preterm (birth)

## Abstract

**Background:** Preterm infants are born with immature organs, leading to morbidities such as necrotizing enterocolitis (NEC), a gut inflammatory disease associated with adverse feeding responses but also hemodynamic and respiratory instability. Skin-to-skin contact including “kangaroo care” may improve infant survival and health *via* improved vital functions (e.g., pulmonary, cardiovascular) and endocrine influences by adrenal glucocorticoids. Clinical effects of skin-to-skin contact for newborn siblings (“co-bedding”) are not known. Using NEC-susceptible Preterm pigs as models, we hypothesized that co-bedding and exogenous glucocorticoids improve vital functions and NEC resistance.

**Methods:** In experiment 1, cesarean-delivered, formula-fed Preterm pigs were reared in incubators with (co-bedding, COB, *n* = 30) or without (single-bedding, SIN, *n* = 29) a sibling until euthanasia and tissue collection on day four. In experiment 2, single-bedded Preterm pigs were treated postnatally with a tapering dose of hydrocortisone (HC, *n* = 19, 1–3 mg/kg/d) or saline (CON, *n* = 19).

**Results:** Co-bedding reduced NEC incidence (38 vs. 65%, *p* < 0.05) and increased the density of colonic goblet cells (+20%, *p* < 0.05) but had no effect on pulmonary and cardiovascular functions (respiration, blood pressure, heart rate, blood gases) or cortisol levels. There were limited differences in intestinal villous architecture and digestive enzyme activities. In experiment 2, HC treatment increased NEC lesions in the small intestine without any effects on pulmonary or cardiovascular functions.

**Conclusion:** Co-bedding may improve gut function and NEC resistance independently of cardiorespiratory function and cortisol levels, but pharmacological cortisol treatment predispose to NEC. Preterm pigs may be a useful tool to better understand the physiological effects of co-bedding, neonatal stressors and their possible interactions with morbidities in Preterm neonates.

## Introduction

Preterm birth (birth before 37 weeks' gestation) interrupts intrauterine development and results in premature exposure of the developing fetus to the extra-uterine environment, leading to a range of morbidities, including necrotizing enterocolitis (NEC). NEC is a life-threatening gut inflammatory disease that develops in part due to dysfunctional barrier and motility of the gut following enteral feeding, especially with formula, and the presence of opportunistic pathogenic bacteria (1). However, physiological stressors and the mesenteric blood supply may also play a role in NEC etiology, exemplified by the reduction in NEC incidence after surgical ligation of patent ductus arteriosus in extremely low birth weight infants (2).

Parent-infant skin-to-skin contact (termed “kangaroo care”) may serve to reduce negative stress effects, thereby increasing cardiovascular and respiratory stability in Preterm infants (3–5). A meta-analysis, including more than 3,000 low birth weight infants, showed that kangaroo care decreased overall mortality and infection rates and increased body weight gain (6). Skin-to-skin contact between infant siblings (termed “co-bedding”) may provide the same benefits as kangaroo care, but few clinical studies are available and effects on survival, vital functions, NEC and infection rates are unknown (7). The mechanisms whereby kangaroo care or co-bedding may improve survival and health remain unknown but could relate to changes in circulating cortisol levels, a key regulator of perinatal organ maturation and cardiovascular and respiratory stability (8). However, the relations among care procedures, circulating cortisol and neonatal morbidities in Preterm infants are not clear (9, 10).

Cortisol is a critical birth adaptation hormone and an essential drug for Preterm infants. This was elegantly demonstrated in the pioneering studies by Liggins et al., incidentally discovering a partial lung expansion in fetal lambs exposed to synthetic cortisol (11), and later showing improved survival and prevention of lung disease in Preterm infants after antenatal betamethasone treatment (12). Besides the beneficial effects on lung maturation, antenatal corticosteroid treatment reduces the risk of NEC (13), but it is unclear whether this is an independent effect or due to improved pulmonary function. Conversely, postnatal corticosteroid therapy does not appear to have organ maturational effects and may even have negative effects on immunity and brain development (e.g., cerebral palsy) (14). Regardless, early postnatal hydrocortisone (HC) treatment is commonly used to support neonatal cardiovascular function and blood pressure (15) and may improve pulmonary function in extremely Preterm infants (16). Effects on gut functions and NEC sensitivity are unknown.

The Preterm pig is a highly translational animal model in experimental neonatology with similarities to moderately Preterm infants in size, physiology, anatomy and presence of a range of relevant physiological immaturities (17). At 80% gestation, Preterm pigs have lower mean arterial blood pressure and require antenatal corticosteroid treatment to survive (17) while at 90% gestation, neither exogenous corticosteroids or mechanical ventilation are strictly required for survival. However, Preterm newborn pigs have lower serum cortisol levels than term counterparts (18), and treatment with adrenocorticotropic hormone (ACTH) or cortisol to pig fetuses or cesarean-delivered near-term pigs improves maturation of the gut, pancreas and liver (19–22). In 90% gestation Preterm pigs, formula feeding alone, without further physiological stressors, is sufficient to induce high sensitivity to develop gut lesions with a high degree of clinical, radiographic and pathological similarity to human NEC (23).

On this background, we hypothesized that neonatal co-bedding and HC treatment are two clinically relevant interventions that together or independently improve cardiovascular and respiratory functions and protect against NEC in Preterm neonates. Using Preterm pigs as models for Preterm infants, we first compared co-housed animals with single-housed siblings and subsequently performed a saline-controlled HC intervention study in single-housed animals. Pulmonary and cardiovascular functions were longitudinally recorded before gut pathological assessment a few days later when Preterm formula-fed pigs are known to be highly NEC sensitive.

## Materials and Methods

### Experiment 1: Co-bedding

**Figure 1:**
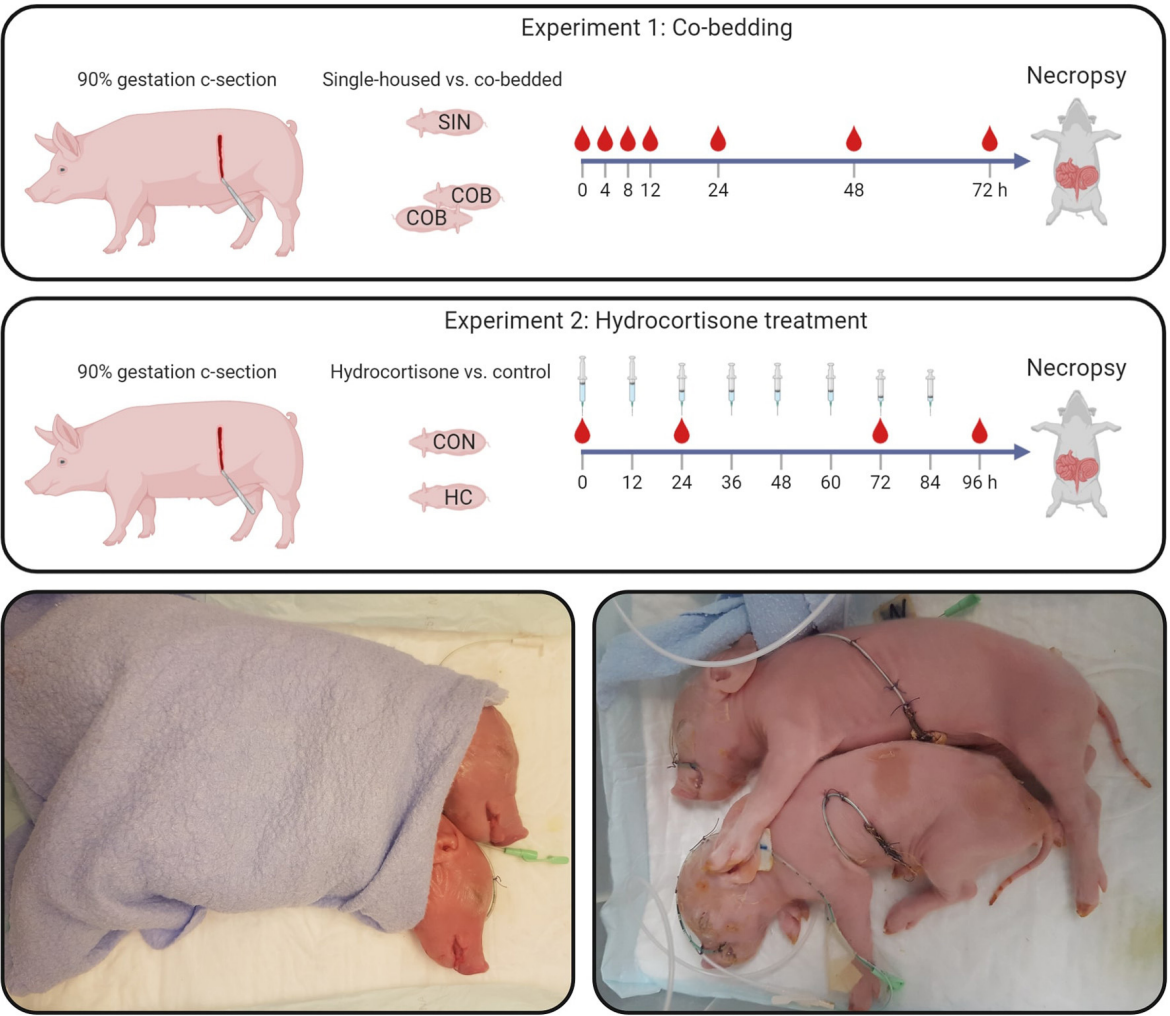
Study design (upper panels). In Experiment 1, single-housed (SIN) and co-bedded (COB) Preterm cesarean-delivered piglets were studied for 72 h, including a period of longitudinal monitoring of vital functions and blood gases. In Experiment 2, Preterm pigs receiving a tapering dose of hydrocortisone (HC) were compared with controls (CON) for 96 h. Main outcomes in both experiments were vital signs, cardiovascular and respiratory functions, as well as gut structure and function, including NEC-like lesions. Lower panels show how co-bedded piglet siblings were wrapped together just after birth and fitted with umbilical catheters and feeding tubes.

Fifty-nine Preterm piglets from three pregnant sows (Danish Landrace × Large White × Duroc) were delivered by cesarean section at 90% gestation (gestational day 106) and resuscitated as previously described (24) (see study design in Figure 1). The newborn pigs were stratified by sex and birth weight and randomly allocated for single-housing (SIN, *n* = 29) or co-bedding (COB, *n* = 30) immediately after birth. Co-bedded animals were paired with a littermate pig and shared a single heated incubator (32–36°C), as shown in Figure 1. In contrast to our standard rearing of Preterm pigs (24), no supplemental oxygen was provided to the incubators within the first 12 h, to allow the piglets to demonstrate their independent ability to reach respiratory stability after some hours. On the first day, co-bedded piglets were wrapped together in a dry cotton cloth, while on subsequent days, they were placed in skin-to-skin contact (Figure 1, bottom panel) whenever they were not walking around in incubators (typically after 24–48 h).

Within 2–3 h after birth, animals were fitted with oro-gastric and umbilical arterial catheters for gavage feeding and vascular access (6 and 4 Fr size, respectively, Portex, Kent, UK), as previously described (24). To compensate for the lack of transplacental immunoglobulin transfer, animals were passively immunized with 16 ml/kg body weight of intra-arterially administered maternal plasma within the first 24 h after birth. The animals were gavage-fed at 3-h intervals with increasing volumes of infant formula (40 ml/kg/d on day 1, increasing to 80 ml/kg/d on day 4–5 (see nutrient compositions in Supplementary Table 1). Throughout the experiment, until euthanasia for tissue collection at ~72 h after birth, the animals were continuously infused with parenteral nutrition *via* the arterial catheter (4 ml/kg/h, Kabiven, Fresenius-Kabi, Bad Homburg, Germany).

### Experiment 2: Hydrocortisone Treatment

Thirty-eight Preterm piglets from two sows were stratified by sex and birth weight and randomly allocated for intra-arterial hydrocortisone injection (HC, *n* = 19; Solu-Cortef, Pfizer, Ballerup, Denmark) or saline (CON, *n* = 19). The tapering dose of HC was 3 mg/kg twice daily on day 1, decreasing to 2 mg/kg on days 2–3, and 1 mg/kg on day 4. The dose regimen was based on clinical HC use for hypotension treatment (25) and prevention of bronchopulmonary dysplasia (26). Animals were euthanized ~96 h after birth. All other procedures were as described above for Experiment 1.

### Vital Signs, Blood Gases, and Cortisol

In the co-bedding experiment, assessment of vital signs and arterial blood sampling for blood gas measurements was done at 0, 4, 8, 12, 24, 48, and 72 h after birth. Serum cortisol levels were measured at 48 and 72 h. In the hydrocortisone experiment, vital signs were recorded at 0, 6, 24, 48, and 72 h after birth, except for arterial blood pressure (only at 24 and 72 h). Arterial blood gases were measured at 24 h, while serum cortisol levels were measured at birth, and at 72 and 96 h (12 h after the final HC administration). Core body temperature was measured using a rectal thermometer. Respiratory frequency was manually counted in 2-min intervals. Heart rate and oxygen saturation were measured using pulse oximetry with the sensor attached to the upper left forelimb. Arterial blood pressure was measured by connecting the umbilical catheter to a saline-filled tubing system pressurized to 200 mmHg and equipped with transducer and monitor (Datex Ohmeda Compact S5, GE Healthcare, Brondby, Denmark). Data was stored and analyzed using Datex-Ohmeda S/5 Collect software. Arterial blood gases were measured using the GEM Premier 3000 system (Intrumentation Laboratory, Bedford, MA, USA). Cortisol levels were measured in serum samples using an enzyme-linked immunosorbent assay (KGE008B, R&D Systems, Abingdon, UK).

### Euthanasia, Tissue Collection, and NEC Evaluation

Three hours prior to euthanasia, all animals received an oro-gastric 15 ml/kg bolus of 5/5% lactulose/mannitol solution for the assessment of intestinal permeability by urinary recovery of the sugar marker molecules. Furthermore, animals in the co-bedding experiment received a standardized oro-gastric formula bolus of 15 mL/kg to assess gastric emptying. Animals were then anesthetized and blood collected by cardiac puncture for hematological cell counting and blood biochemistry (only for the HC experiment) measurements, followed by euthanasia with a lethal cardiac injection of barbiturate. Urine was collected by cystocentesis for measurement of lactulose and mannitol, as previously described (27). Abdominal and thoracic organs were excised and brains collected for recording of their wet weights. Macroscopic lesions of small intestine and colon were graded according to a previously validated six-grade NEC scoring system (see Supplementary Figure 1), where NEC was defined as the presence of a lesion score of three (focal hemorrhage) or above in any of the gut segments (28). Biopsies of proximal, mid and distal small intestine were dissected and either immersion fixed to assess mucosal morphometry or snap-frozen for later measurement of brush-border enzyme activity and oxidative stress markers (only for the HC experiment), while a biopsy from the transverse colon was collected and immersion fixed for estimation of goblet cell mucin density (only for the co-bedding experiment).

### Gut Structure and Function

Duplicate biopsies from three small intestinal regions were paraffin-embedded, and tissue sections stained with hematoxylin-eosin. Villus lengths and crypt depths were measured with ImageJ software in four 10× magnification photomicrographs per region capturing mainly well-oriented villus structures. Further, duplicate biopsies from transverse colon were embedded in paraffin, and tissue sections stained with Alcian blue-periodic acid Schiff to visualize mucin-laden goblet cells. Relative goblet cell area was quantified using ImageJ software. Small intestinal brush-border enzyme activities were measured as previously described (29). Finally, markers of oxidative status in small intestinal homogenate were measured. Superoxide dismutase (SOD) acticity was analyzed using the Ransod colorimetric assay (Randox, Crumlin, UK). Oxidized (GSSG) and reduced (GSH) glutathione were analyzed by a fluorometric assay (30), Vitamin E (α- and γ-tocopherol), ascorbic acid and malondialdehyde (MDA), a lipid peroxidation end product, were analyzed by high-performance liquid chromatography (31–33).

### Statistics

All statistical analyses were performed using R statistical software (version 1.3.1093). Analysis of dichotomous outcomes (NEC and diarrhea incidences) was done using a generalized linear model (glm), while continuous outcomes were analyzed using a general linear model (lm). Longitudinal data (vital functions, blood gases, mucosa morphometry) were analyzed using a linear mixed-effects (lme) model with animal ID as a random effect parameter. All models included adjustments for litter, sex and birth weight as fixed-effects parameters. Residuals were checked using Q-Q and residual vs. fit plots and data log-transformed, if these did not adhere to model assumptions of normality and homoscedasticity. Probability values <0.05 were considered significant.

## Results

### Experiment 1: Co-bedding

**Figure 2:**
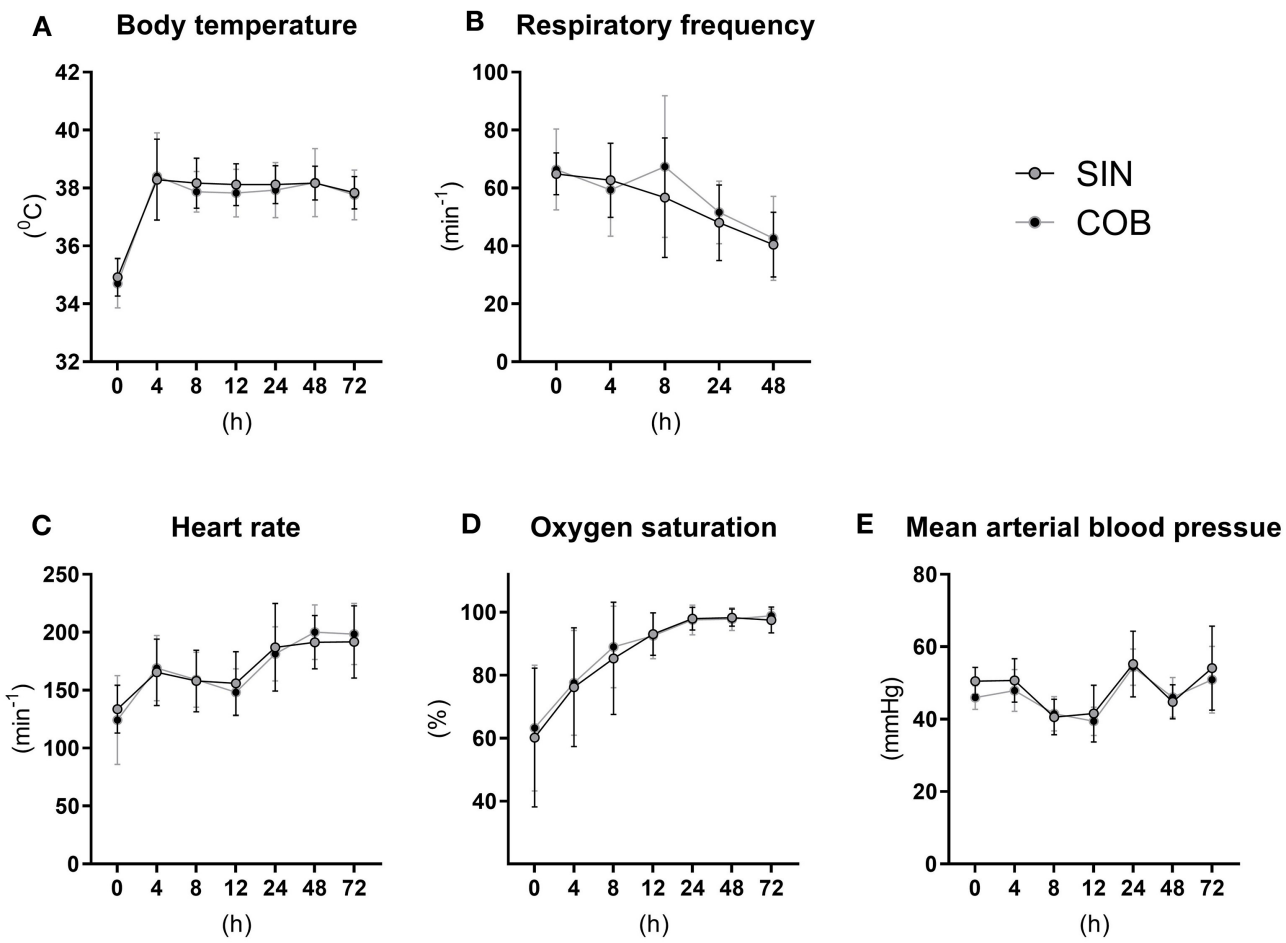
Core body temperature **(A)** and pulmonary and cardiovascular functions **(B–E)** in single-housed (SIN) and co-bedded (COB) Preterm piglets. Data is presented as means with standard deviations.

The vital functions of co-bedded and single-housed Preterm piglets were closely monitored for 72 h (Figure 2). Following transient hypothermia in the hours following Preterm birth, the core body temperature increased to normal levels in both groups (38–39°C) within 4 h (Figure 2A). The respiratory frequency decreased gradually, and similarly in the groups, together with increasing heart rate and oxygen saturation (Figures 2B–D). Large intra- and inter-individual variation (e.g., 40–50 mmHg) were observed in mean arterial blood pressures with no obvious effect of time or treatment (Figure 2E).

**Figure 3:**
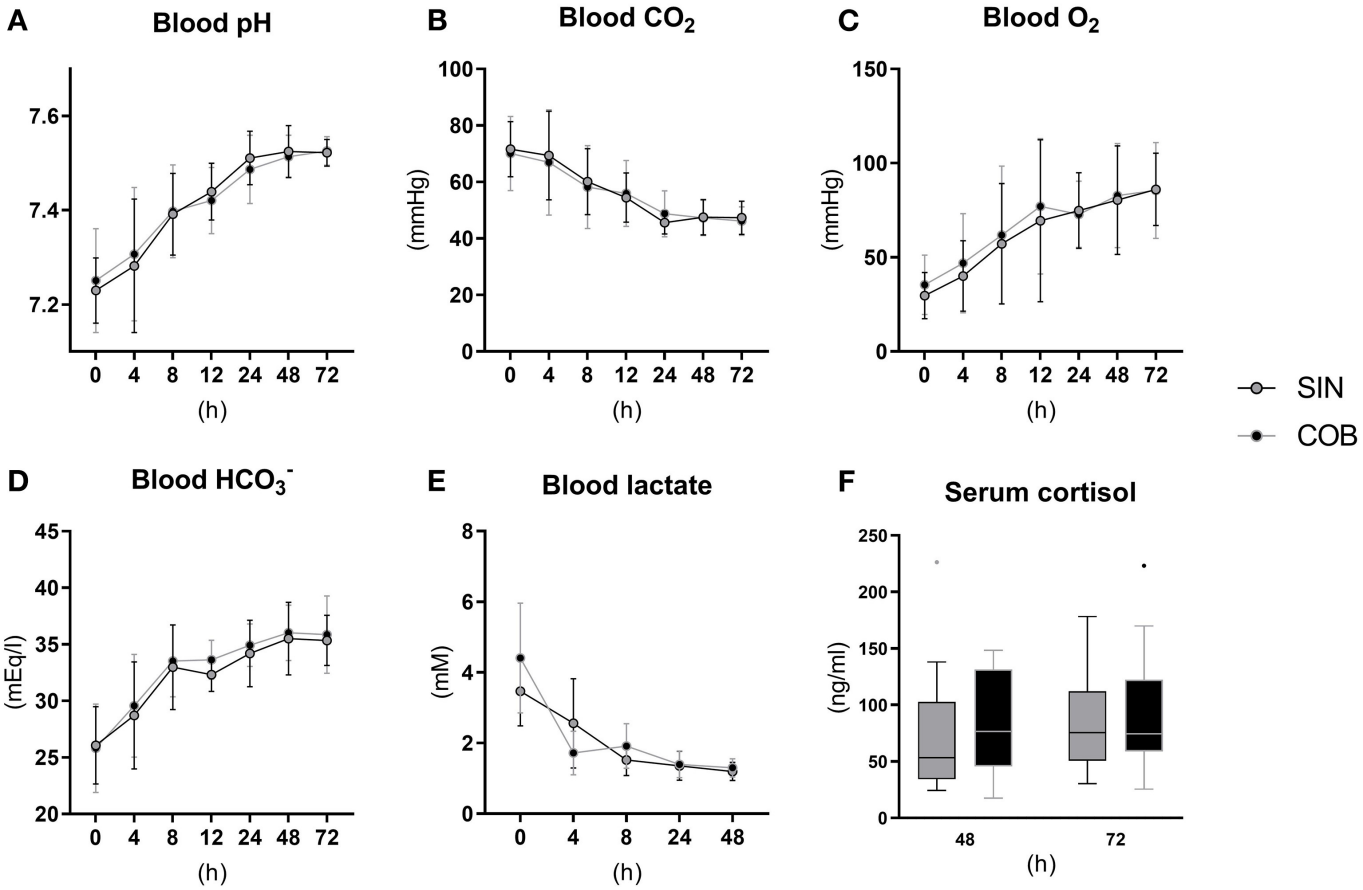
Arterial blood gas values **(A–E)** and serum cortisol levels **(F)** in single-housed (SIN) and co-bedded (COB) Preterm piglets. Longitudinal data is presented as means with standard deviations, and cortisol data is presented in box plots (box edges denote interquartile ranges and horizontal line denotes the median value) with Tukey whiskers (outliers shown as dots).

Pronounced changes over time were also observed for arterial blood gas parameters (Figures 3A–E), measured at similar time points as the vital signs. Piglets were mildly acidotic just after birth (mean arterial blood pH around 7.2, Figure 3A) but blood acidity was normalized within the first 24 h, and similarly in the two groups. Accordingly, CO_2_ partial pressure (Figure 3B) and lactate levels decreased over time (Figure 3E), whereas O_2_ partial pressure and bicarbonate levels increased (Figures 3C,D), with no effect of co-bedding vs. single-bedding. Likewise, serum cortisol levels measured at 48 and 72 h (Figure 3F), and blood hematology at 72 h (Table 1), showed no differences between the groups.

**Table 1:** Blood hematology of three-day old single-housed or co-bedded piglets.

	**SIN**	**COB**
Total erythrocytes (10^12^/l)	3.79 (0.57)	3.66 (0.49)
Hemoglobin (mmol/l)	4.92 (0.62)	4.75 (0.46)
Hematocrit (g/kg)	0.26 (0.03)	0.25 (0.03)
Platelets (10^9^/l)	276 (46.5)	233 (33.2)
Total leucocytes (10^9^/l)	1.78 (1.04)	1.44 (0.76)
Neutrophils (10^9^/l)	0.79 (0.83)	0.68 (0.68)
Lymphocytes (10^9^/l)	0.77 (0.26)	0.66 (0.15)
Monocytes (10^9^/l)	0.07 (0.10)	0.05 (0.02)

*All data is presented as means with standard deviations in parentheses. SIN, single-housing; COB, co-bedding. No differences between the groups were found in the statistical analysis*.

**Figure 4:**
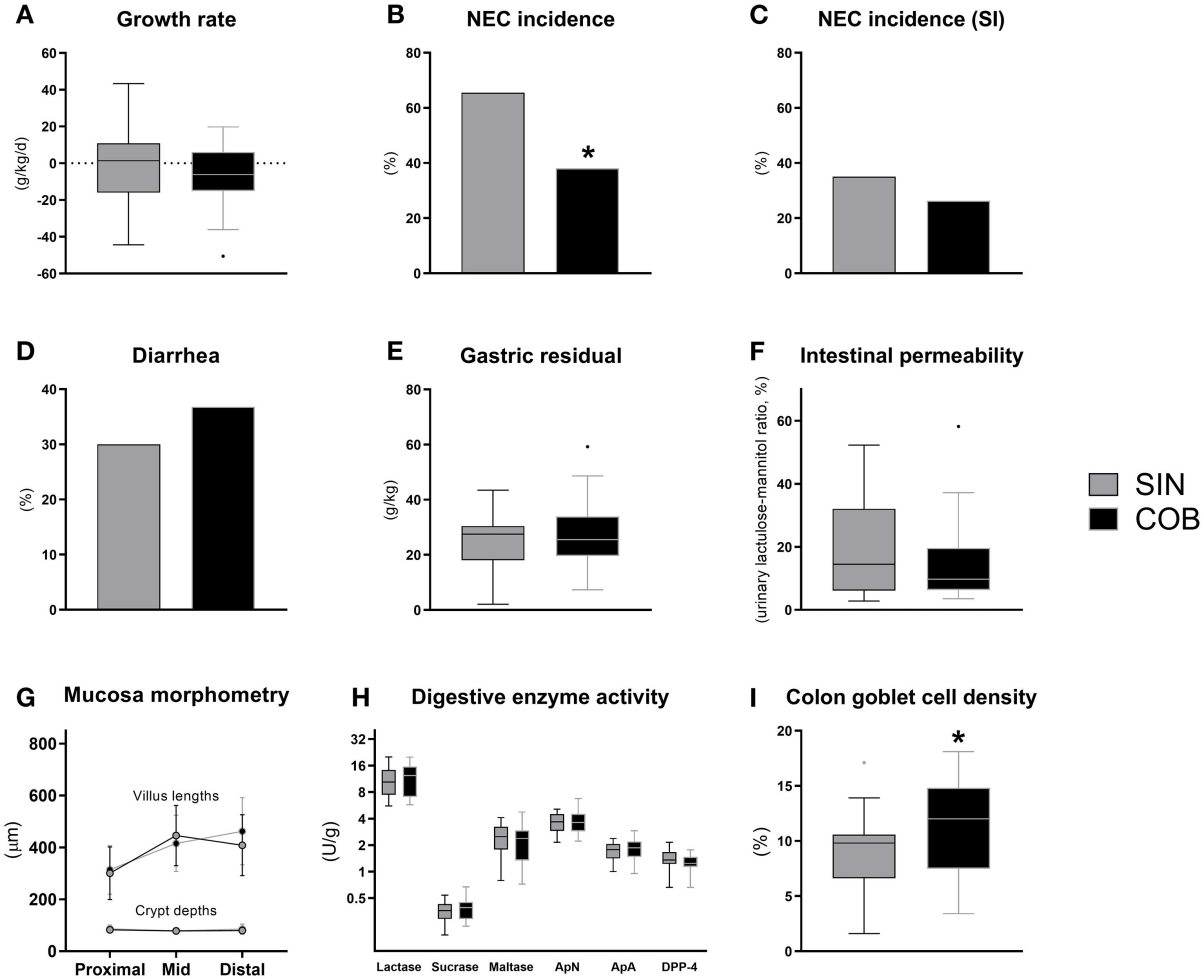
Body growth rate **(A)**, overall and small intestinal (SI) NEC incidence **(B,C)**, proportion of pigs with diarrhea **(D)**, gastric residual volume **(E)**, urinary lactulose-mannitol ratio as a measure of intestinal permeability **(F)**, small intestinal villus and crypt morphology **(G)**, activity of brush-border disaccharidases (lactase, sucrase, maltase) and aminopeptidases (aminopeptidase N, ApN; aminopeptidase A, ApA; dipeptidyl peptidase-4, DPP-4) **(H)**, and relative area of colonic goblet cells **(I)**. Dichotomous data is reported as frequencies. Longitudinal data is presented as means with standard deviations, while remaining continuous data is presented as box plots (box edges denote interquartile ranges and horizontal line denotes the median value) with Tukey whiskers (outliers shown as dots). *Denotes a probability value below 0.05. SIN, single-housing; COB, co-bedding.

Until euthanasia at 72 h, daily growth rate was similar between the groups (Figure 4A) and no differences were observed for organ weights (Supplementary Table 2). At this time, the NEC incidence was significantly lower in co-bedded animals relative to single-housed littermates (*p* < 0.05, Figure 4B). This was due to a reduction in colonic NEC-like lesions, as the small intestinal NEC incidences were similar (Figure 4C). Conversely, diarrhea incidence, gastric emptying rate and intestinal permeability (Figures 4D–F) were not affected by co-bedding. Likewise, small intestinal villi and crypts were of similar lengths (Figure 4G), and disaccharidase and aminopeptidase activities did not differ (Figure 4H). However, supporting the reduced colonic NEC incidence, significantly higher colonic goblet cell density was observed in co-bedded animals (*p* < 0.05, Figure 4I).

### Experiment 2: Hydrocortisone Treatment

**Figure 5:**
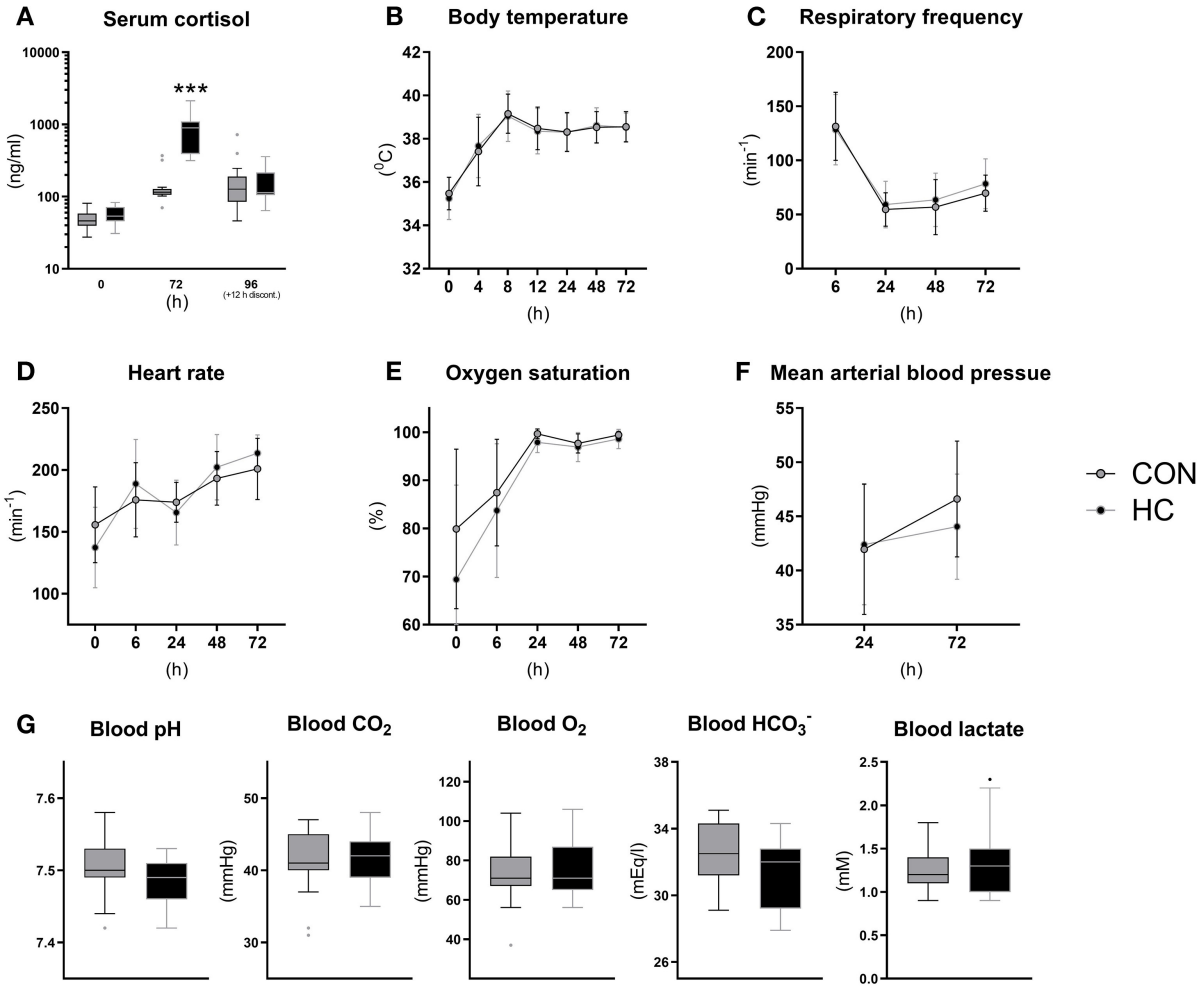
Serum cortisol levels **(A)**, core body temperature **(B)**, pulmonary and cardiovascular functions **(C–F)** and arterial blood gas parameters at 24 h **(G)** for hydrocortisone (HC) and control (CON) Preterm piglets. Longitudinal data is presented as means with standard deviations, and remaining data is presented as box plots (box edges denote interquartile ranges and horizontal line denotes the median value) with Tukey whiskers (outliers shown as dots). ^***^Denotes a probability value below 0.001.

Following the tapering HC dose over 4 days, serum cortisol levels were measured at birth and 72 h (shortly after HC administration) and at euthanasia (~12 h after the last HC injection (Figure 5A). Cortisol levels at birth were ~50 ng/ml on average and increased to a concentration just above 100 ng/ml at 72 and 96 h in CON animals. Cortisol levels were more than six-fold higher in HC animals relative to CON at 72 h (*p* < 0.001) but appeared to decrease to control levels 12 h after treatment discontinuation (Figure 5A). Vital functions followed a similar longitudinal trend as observed in Experiment 1, with increasing core body temperature, pulse and oxygen saturation (Figures 5B,D,E) and decreasing respiratory frequency (Figure 5C). Like in Experiment 1, all blood gas values were largely normalized by 24 h after birth, and there were no clear effect of HC treatment on these (Figure 5G) or on blood pressure (Figure 5F). In addition, blood hematology (Supplementary Table 3) and biochemical profiles (Supplementary Table 4) measured at euthanasia showed no effects of HC treatment.

**Figure 6:**
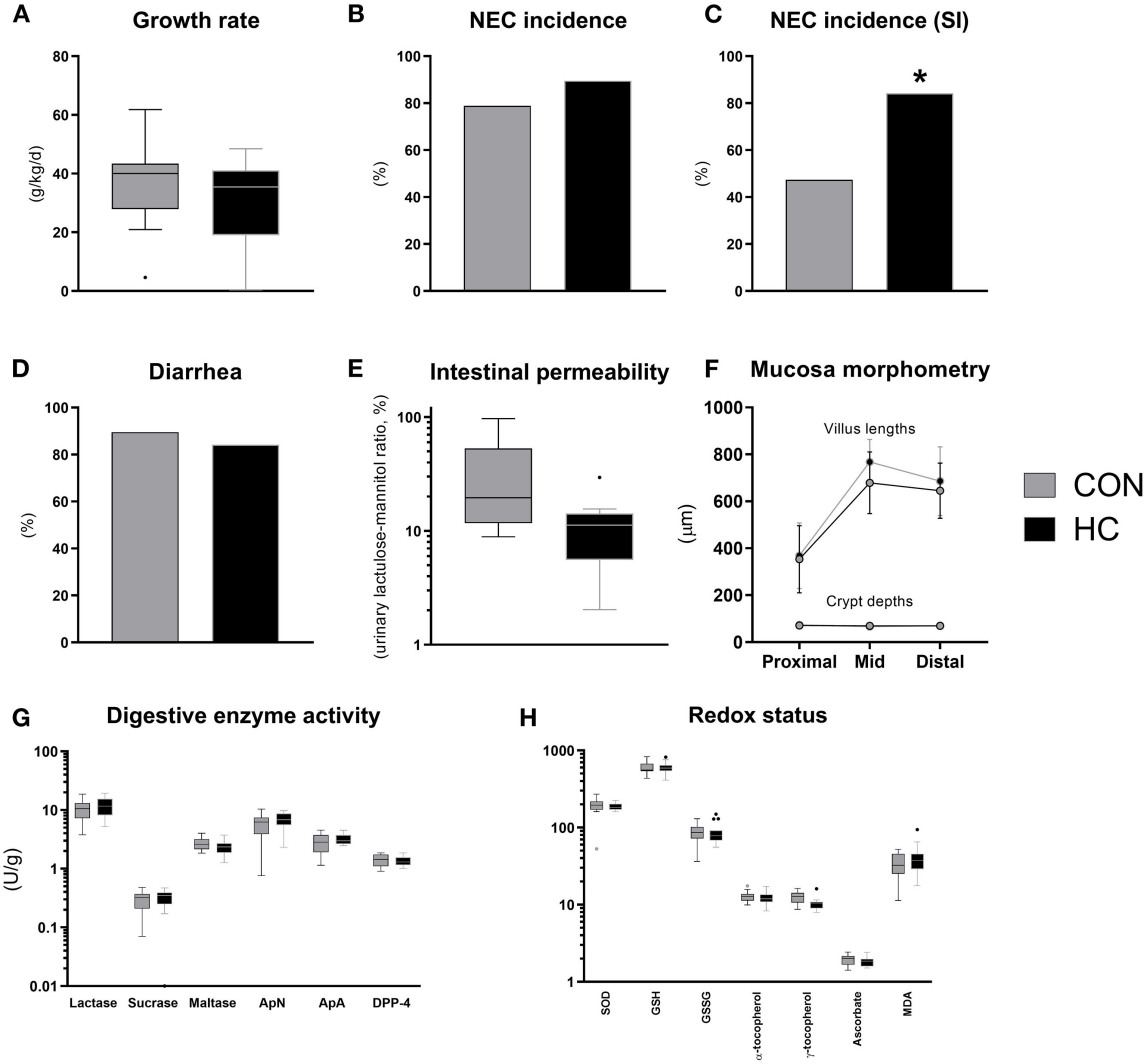
Body growth rate **(A)**, overall and small intestinal (SI) NEC incidence **(B,C)**, proportion of pigs with diarrhea **(D)**, urinary lactulose-mannitol ratio as a measure of intestinal permeability **(E)**, small intestinal villus and crypt morphology **(F)**, activity of brush-border disaccharidases (lactase, sucrase, maltase) and aminopeptidases (aminopeptidase N, ApN; aminopeptidase A, ApA; dipeptidyl peptidase-4, DPP-4) **(G)**, markers of oxidative stress in small intestinal tissue (superoxide dismutase activity, SOD, U/g; reduced (GSH) and oxidized glutathione, GSSG, both nmol/g; α- and γ-tocopherol, both nmol/g; ascorbate, μmol/g; malondialdehyde, MDA, nmol/g) **(H)**. Dichotomous data is reported as frequencies. Longitudinal data is presented as means with standard deviations, while remaining continuous data is presented as box plots (box edges denote interquartile ranges and horizontal line denotes the median value) with Tukey whiskers (outliers shown as dots). ^*^Denotes a probability value below 0.05. HC, hydrocortisone; CON, control.

Until euthanasia at 96 h after birth, daily growth rate was not affected by HC treatment (Figure 6A) and organ weights did not differ (Supplementary Table 5). The incidence of small intestinal NEC lesions was significantly increased in the HC group, relative to CON (*p* < 0.05), without effects on the overall NEC incidence across gut regions (Figures 6B,C). The incidence of diarrhea was similarly high in both groups (Figure 6D), while the HC group showed a tendency to reduced intestinal permeability (Figure 6E, *P* = 0.07). Further, no treatment effects were observed for small intestinal villous architecture, brush-border enzyme activities and markers of oxidative status (Figures 6F–H). However, across groups animals with NEC showed increased MDA levels relative to littermates without NEC (*p* < 0.05, data not shown).

## Discussion

With increasing survival of extremely Preterm infants, improving the care for these patients remains a priority to facilitate optimal growth and development and reduce the risk of serious morbidities. Skin-to-skin contact appears to provide organism-wide benefits to Preterm infants, but detailed knowledge about physiological effects requires the use of animal models. With the use of Preterm pigs, we examined the potential of Preterm sibling skin-to-skin contact (co-bedding) as well as pharmacological cortisol treatment to improve vital functions and prevent the life-threatening disease NEC. Cardiovascular and respiratory function were not notably affected by either treatment but co-bedding decreased overall NEC incidence while HC treatment increased NEC specifically in the small intestine. The two experiments provide preliminary insights into some clinically relevant interventions for Preterm infants but more studies are required to better understand mechanisms and possible interactions across organs and functions.

Co-bedding and kangaroo care share the concept of skin-to-skin contact and possible effects on neuro-endocrine regulatory pathways, but they also differ in many aspects. The majority of clinical data on skin-to-skin clinical care in Preterm infants is based on kangaroo care, documented to increase survival, growth and infection resistance (6), in addition to possible effects on the oral and stool microbiome (34, 35). The latter effect may arise from exposure to maternal skin microbiota, a factor that we have excluded in our co-bedding mediated skin-to-skin experiments. Growth and feeding results from studies of kangaroo care should also be interpreted with caution, as it is often difficult to differentiate between the effects of breastmilk intake and skin-to-skin contact *per se*. In this perspective, it is attractive from a scientific standpoint to use cesarean-delivered (sterile) Preterm pigs, not affected by prenatal complications, and fed without contact to their mother in highly standardized conditions, in studies on skin-to-skin contact *via* siblings, affecting relevant physiological, clinical and pathological endpoints.

Across all mammals, organ structure and function develop differently in relation to the normal time of birth (24, 36). In pigs, gut and immune functions are relatively immature until close to term, making them highly susceptible to gut dysfunctions (e.g., NEC) and infections when delivered at 90% gestation (37, 38). For these pigs, the development in vital organ functions just after birth was characterized by a transient hypothermia, tachypnea, low oxygen saturation and blood pH as well as elevated lactate but all these parameters resolved to normal levels within 24–48 h after birth when pigs were kept in heated incubators with fluid and nutrition supply (parenteral nutrition), and even without oxygen supplementation. In this regard, neonatal viability of 90% gestation Preterm pigs may reflect that in moderately Preterm infants (32–37 weeks gestation), rather than very or extreme Preterm infants (<32 weeks gestation). This is supported by the limited need for surfactant, oxygen and mechanical ventilation for survival of 90% gestation Preterm pigs. A large proportion of these pigs have an open ductus arteriosus (PDA) at 24 h after birth, relative to term pigs (20–40 vs. 0%, our unpublished observations). In addition to a moderate cardiovascular and respiratory immaturity in 90% gestation Preterm pigs, anesthesia of the pregnant sow during the cesarean section, could also have contributed to the compromised physiological state in the first day of life. The fact that normalization of vital functions during the first 1–2 days was not accelerated by co-bedding or HC treatment may indicate that such interventions are most pronounced in more extreme states of prematurity.

Antenatal glucocorticoid treatment is among the most well-documented medical treatments for Preterm infants with beneficial effects on survival and pulmonary function (13). After Preterm birth on the other hand, it has proven difficult to identify the proper drug and dose regimen to patients with insufficient endogenous glucocorticoid production to ensure optimal clinical benefit (e.g., treatment of hypotension) without adverse treatment effects (14). A frequent side effect to postnatal glucocorticoid treatment is gastrointestinal bleeding (14). In this study, we found an increased incidence of small intestinal hemorrhage (NEC-like lesions) in HC treated Preterm pigs, which may be due to excessive circulating cortisol levels. Oxidative tissue damage was confirmed by increased levels of MDA, a lipid peroxidation product shown in animals with hemorrhagic small intestine. Establishing reference values for serum cortisol levels in Preterm infants is difficult due to fluctuations and numerous influencing factors (e.g., antenatal glucocorticoids, delivery mode, glucose levels, infection). However, 20–200 ng/ml has been proposed as a relatively wide range reflecting cortisol levels in non-symptomatic Preterm infants (39) and is in accordance with that of Preterm pigs in the neonatal period (40). In this regard, co-bedded and single-housed Preterm pigs had cortisol levels in the normal range, whereas HC treated animals largely exceeded these reference values. The initial HC dose used in this study (3 mg/kg/d) is three-fold higher than the dose that has been shown to be safe and efficacious for prevention of bronchopulmonary dysplasia in Preterm infants (16). In summary, 90% gestation pigs may not benefit from exogenous cortisol postnatally with regards to their cardiaovascular and respiratory functions, potentially because these functions are relatively mature. This contrasts the marked effects of ACTH or HC treatment on gut functions in both near-term or term pigs (20, 21).

We found that skin-to-skin contact between Preterm newborn pigs increased the resistance to NEC-like colonic inflammation in ways that are unlikely to depend on cardiopulmonary functions or cortisol levels. The benefits of co-bedding on NEC was confined to the colon region, which showed less NEC-like pathology and higher relative area of goblet cells in co-bedded animals. The suggestion that Preterm skin-to-skin contact improves stress coping *via* neuroendocrine mechanisms (41) may help to explain how co-bedding could reduce NEC incidence without affecting vital functions. Heart rate variability, an accepted marker for cardiac vagal tone and parasympathetic nervous activity, is altered in Preterm relative to term infants (42), and reduced by kangaroo care in response to pain (43). Further, massage of Preterm infants, another type of skin-to-skin contact, improves gastric motility in addition to increasing vagal tone. Whereas increases in vagal tone is usually accompanied by decreased cortisol levels, evidence from inflammatory bowel disease patients indicates that this inverse association may be uncoupled during disease states (44). The gut microbiota is closely associated with gut inflammatory status and NEC, but to which degree it is affected by co-bedding and whether this is causally related with NEC development remains to be explored. To address this issue, we propose a future experiment, where gut microbiota from co-bedded Preterm neonates is transplanted into single-housed recipients to investigate causality of the gut microbiota in this regard. Collectively, we speculate that skin-to-skin contact during co-bedding increases vagal tone, thereby promoting gastrointestinal motility and improves NEC resistance, possibly through modulation of the gut microbiota.

Kangaroo care has become standard practice in most neonatal clinics, but in situations where a parent is not able to be in contact with the infant (e.g., culture, tradition, socioeconomic reasons), co-bedding may provide an alternative, even among non-siblings if deemed safe. Regardless, the causal relationships among skin-to-skin contact, vagal tone, gastrointestinal motility and inflammation clearly warrants further investigations and the Preterm pig may be of value to in this context to study detailed effects of clinical care procedures.

## Data Availability Statement

The raw data supporting the conclusions of this article will be made available by the authors, without undue reservation.

## Ethics Statement

All *in vivo* procedures were approved by the Danish Animal Experiments Inspectorate (license no. 2020-15-0201-00520), which complies with the EU Directive 2010/63 (legislation for the use of animals in research).

## Author Contributions

AB and PTS: conception and design, data interpretation, writing original draft, and critical review and editing. AB and YP: data acquisition. AB, YP, YL, and JL: data analysis. All authors: editing and approval of the final manuscript.

## Conflict of Interest

The authors declare that the research was conducted in the absence of any commercial or financial relationships that could be construed as a potential conflict of interest.
