# Supplementary material for: Co-bedding of Preterm Newborn Pigs Reduces Necrotizing Enterocolitis Incidence Independent of Vital Functions and Cortisol Levels
Source: Front Pediatr. 2021 Apr 1;9:636638. doi: 10.3389/fped.2021.636638 (PMC8049114; doi:10.3389/fped.2021.636638)
Supplement: Supplementary file 1 [file Data_Sheet_1.docx]

**SUPPLEMENTARY MATERIAL**

**Suppl. figure S1.** Gut pathological scoring criteria


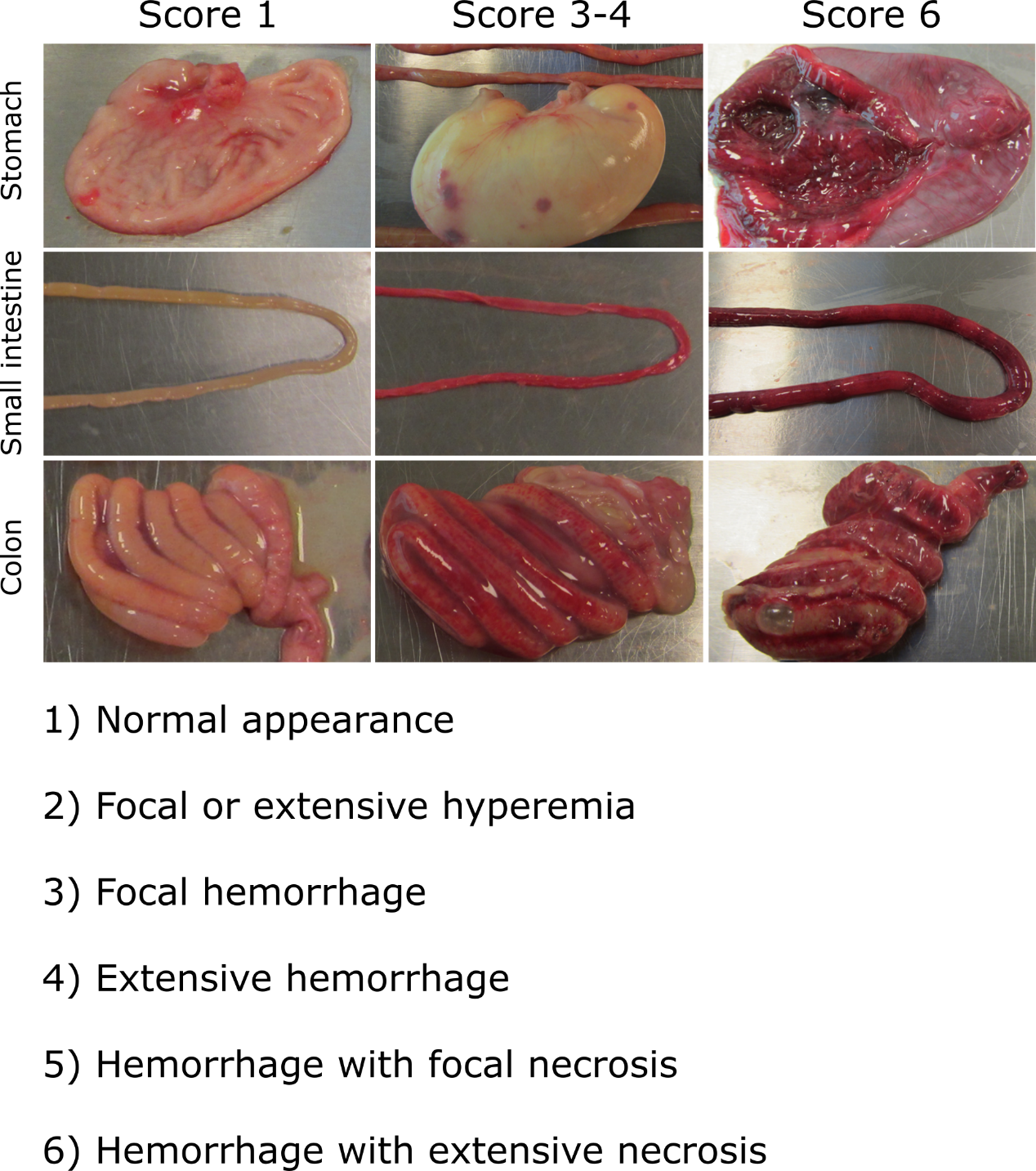


**Suppl. table S1.** Infant formula compositions

|  | **Co-bedding experiment** | **Hydrocortisone experiment** |
| --- | --- | --- |
|  | Formula constituents | |
|  | 20 g/l Variolac 960 * | Enfamil premature lipil ready-to-drink formula ^†^ |
|  | 20 g/l Fantomalt ^#^ |  |
|  | 15 g/l Miprodan 40 * |  |
|  | 55 g/l Lacprodan DI9224 * |  |
|  | 70 g/l Liquigen MCT ^#^ |  |
|  | 20 g/l Calogen LCT ^#^ |  |
|  | 2 g/l Phlexyvits ^#^ |  |
|  |  |  |
|  | Nutrient composition | |
| Energy (kJ/l) | 3317 | 3400 |
| Protein (g/l) | 62 | 24 |
| Lipid (g/l) | 45 | 41 |
| Carbohydrate (g/l) | 36 | 89 |
| Sugars (g/l) | 19 | 36 |
| ^#^ Manufactured by Nutricia, Allerod, Denmark. Manufactured by Arla Foods Ingredients, Viby J, Denmark. ^†^ Manufactured by Mead-Johnson Nutrition (Chicago, IL, USA) | | |

**Suppl. table S2.** Organ weights of three-day old single-housed or co-bedded piglets.

|  | **SIN** | **COB** | |
| --- | --- | --- | --- |
| Small intestine (g/kg) | 24.6 (3.07) | 25.6 (4.52) | |
| Colon (g/kg) | 14.9 (3.97) | 15.8 (6.81) | |
| Liver (g/kg) | 24.7 (4.51) | 27.5 (6.51) | |
| Spleen (g/kg) | 2.78 (4.17) | 1.89 (0.34) | |
| Heart (g/kg) | 7.56 (1.17) | 8.31 (1.80) | |
| Lungs (g/kg) | 29.0 (7.14) | 61.3 (8.57) | |
| Kidneys (g/kg) | 10.2 (1.45) | 9.66 (2.42) | |
| Adrenal glands (g/kg) | 0.24 (0.07) | 0.24 (0.08) | |
| Cerebrum (g) | 23.5 (2.12) | 23.3 (1.99) | |
| Cerebellum (g) | 2.55 (0.22) | 2.57 (0.23) | |
| Brain stem (g) | 2.75 (0.17) | 2.69 (0.20) | |
| All data is presented as means with standard deviations in parentheses. SIN, single-housing; COB, co-bedding. No differences between the groups were found in the statistical analysis. | | |  |

**Suppl. table S3.** Blood hematology of four-day old hydrocortisone treated and control piglets.

|  | **CON** | **HC** |
| --- | --- | --- |
| Total erythrocytes (10^12^/l) | 3.73 (0.60) | 3.74 (0.46) |
| Hemoglobin (mmol/l) | 4.71 (0.76) | 4.71 (0.59) |
| Hematocrit (g/kg) | 0.25 (0.04) | 0.25 (0.03) |
| Platelets (10^9^/l) | 92.2 (34.8) | 103 (24.5) |
| Total leucocytes (10^9^/l) | 1.93 (1.00) | 2.09 (0.89) |
| Neutrophils (10^9^/l) | 0.81 (0.61) | 1.09 (0.65) |
| Lymphocytes (10^9^/l) | 1.00 (0.48) | 0.88 (0.46) |
| Monocytes (10^9^/l) | 0.07 (0.07) | 0.07 (0.05) |
| All data is presented as means with standard deviations in parentheses. CON, control; HC, hydrocortisone. No differences between the groups were found in the statistical analysis. | | |

**Suppl. table S4.** Blood biochemistry of four-day old hydrocortisone treated and control piglets.

|  | **CON** | **HC** |
| --- | --- | --- |
| Albumin (g/l) | 12.0 (2.12) | 12.2 (1.58) |
| Total protein (g/l) | 29.8 (4.40) | 29.8 (2.38) |
| Alkaline phosphatase (U/l) | 3321 (939) | 2897 (740) |
| ALAT (U/l) | 17.3 (5.37) | 14.5 (1.71) |
| ASAT (U/l) | 46.1 (74.8) | 35.3 (25.4) |
| Total bilirubin (µM) | 3.48 (1.50) | 3.13 (1.10) |
| Total cholesterol (mM) | 1.87 (0.54) | 1.90 (0.31) |
| Creatinine (µM) | 53.9 (14.5) | 58.0 (15.2) |
| Creatine kinase (U/ml) | 163 (183) | 124 (80.8) |
| Total bile acids (µM) | 12.3 (9.99) | 21.6 (18.2) |
| Amylase (U/l) | 1305 (233) | 1306 (319) |
| Blood urea nitrogen (mM) | 1.53 (1.47) | 1.82 (0.92) |
| GGT (U/l) | 27.8 (14.1) | 23.9 (14.7) |
| Phosphate (mM) | 1.20 (0.28) | 1.29 (0.32) |
| Calcium (mM) | 2.92 (0.27) | 2.97 (0.20) |
| Magnesium (mM) | 0.87 (0.10) | 0.91 (0.11) |
| Sodium (mM) | 148 (4.66) | 150 (5.58) |
| Potassium (mM) | 4.36 (0.68) | 4.32 (0.53) |
| All data is presented as means with standard deviations in parentheses. CON, control; HC, hydrocortisone. ALAT, alanine-aminotransferase; ASAT, aspartate-aminotransferase; GGT, gamma-glutamyl transferase. No differences between the groups were found in the statistical analysis. | | |

**Suppl. table S5.** Organ weights of four-day old hydrocortisone treated and control piglets.

|  | **CON** | **HC** | |
| --- | --- | --- | --- |
| Small intestine (g/kg) | 26.6 (3.82) | 28.2 (2.75) | |
| Colon (g/kg) | 8.23 (1.66) | 8.89 (1.89) | |
| Liver (g/kg) | 37.4 (3.62) | 35.7 (3.11) | |
| Spleen (g/kg) | 2.17 (0.52) | 1.78 (0.49) | |
| Heart (g/kg) | 7.59 (1.03) | 7.23 (0.91) | |
| Lungs (g/kg) | 20.8 (2.93) | 21.6 (4.07) | |
| Kidneys (g/kg) | 7.84 (1.09) | 8.12 (1.12) | |
| Adrenal glands (g/kg) | 0.17 (0.03) | 0.17 (0.05) | |
| Cerebrum (g) | 23.2 (0.92) | 24.3 (1.48) | |
| Cerebellum (g) | 2.59 (0.15) | 2.75 (0.21) | |
| Brain stem (g) | 2.58 (0.14) | 2.68 (0.18) | |
| All data is presented as means with standard deviations in parentheses. CON, control; HC, hydrocortisone. No differences between the groups were found in the statistical analysis. | | |  |
